# A Rapid and Facile Approach for the Recycling of High‐Performance LiNi_1−*x*−*y*_Co_*x*_Mn_*y*_O_2_ Active Materials

**DOI:** 10.1002/cssc.202001915

**Published:** 2020-09-10

**Authors:** Jan O. Binder, Sean P. Culver, Wolfgang G. Zeier, Jürgen Janek

**Affiliations:** ^1^ Institute of Physical Chemistry Justus-Liebig-University Giessen Heinrich-Buff-Ring 17 35392 Giessen Germany; ^2^ Center for Materials Research (LaMa) Justus-Liebig-University Giessen Heinrich-Buff-Ring 16 35392 Giessen Germany; ^3^ Institute for Inorganic and Analytical Chemistry University of Muenster Correnstrasse 30 48149 Münster Germany

**Keywords:** cathode active material, energy storage, LiNi_0.8_Co_0.1_Mn_0.1_O_2_, lithium-ion battery, recycling

## Abstract

The demand for lithium‐ion batteries has risen dramatically over the years. Unfortunately, many of the essential component materials, such as cobalt and lithium, are both costly and of limited abundance. For this reason, the recycling of lithium‐ion battery electrodes is crucial to ensuring the availability of such resources and protecting the environment. Herein, a simple and scalable recycling process was developed for the prototypical cathode active material Li_1.02_(Ni_0.8_Co_0.1_Mn_0.1_)_0.98_O_2_ (NCM‐811). By a combination of thermal decomposition and dissolution steps, spent NCM could be converted into Li_2_CO_3_ and a transition metal oxalate blend, which served as precursors for new NCM. Importantly, it was also possible to individually separate each transition metal during the recycling process, thereby extending the utility of this method to a wide variety of NCM compositions. Each intermediate in the process was investigated by scanning electron microscopy and X‐ray diffraction. Additionally, the elemental composition of the recycled NCM‐811 was confirmed using inductively coupled plasma optical emission spectroscopy and energy‐dispersive X‐ray spectroscopy. The electrochemical performance of the recycled NCM‐811 exhibited up to 80 % of the initial capacity of pristine NCM‐811. The method presented herein serves as an efficient and environmentally benign alternative to existing recycling methods for lithium‐ion battery electrode materials.

## Graphical abstract

**Recycle and re‐synthesize**: Spent LiNi_0.8_Co_0.1_Mn_0.1_O_2_ (NCM‐811) from Li‐ion batteries is recovered by a facile and inexpensive recycling approach. After a series of decomposition and dissolution reactions, Ni/Co/MnC_2_O_4_ and Li_2_CO_3_ are formed, which serve as precursors for the re‐synthesis of NCM‐811. Additionally, the individual separation of the transition metal compounds is possible.

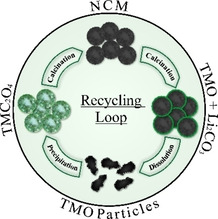

## Introduction

Lithium‐ion batteries are the most pervasive energy storage technology in recent times. In addition to the extensive employment of lithium‐ion batteries in many small‐scale commercial electronics, the development of electric vehicles has bolstered the demand within the automotive industry as well. Notably, the exceptional energy and power densities exhibited by these battery systems arise from the high‐performance electrode materials, for example, graphite at the anode and LiNi_1−*x*−*y*_Co_*x*_Mn_*y*_O_2_ (NCM) at the cathode. In particular, the cathode active material LiNi_0.8_Co_0.1_Mn_0.1_O_2_ possesses a large theoretical capacity of over 270 mAh g^−1^, along with a high mean working voltage of approximately 3.8 V vs. Li^+^/Li.^[1]^ Nevertheless, due to the high cost and decreasing abundance of essential elements used in the production of NCM, the development of recycling processes to recover and reuse the elemental components is imperative.^[2]^

Several recycling approaches can already be found in the literature, employing a variety of different techniques, such as hydrometallurgical processes, direct regeneration, and electrochemical recycling, among others.[^3^, ^4^, ^5^, ^6^, ^7^, ^8^, ^9^, ^10^, ^11^, ^12^, ^13^, ^14^, ^15^, ^16^, ^17^] The most common approaches are hydrometallurgical, given the availability of a large‐scale processing infrastructure, which is already in use for the extraction of metals from their ores. Nevertheless, such methods suffer from long process chains due to the need for separate extraction procedures for each metal.[^9^, ^18^] Another interesting approach is the direct regeneration of active materials.[^3^, ^19^] Recently, a sophisticated approach has been presented by Meng et al.^[20]^ By ball milling a blend of spent NCM and Li_2_CO_3_ followed by a sintering step, a mechanochemical regeneration was achieved.^[20]^ Yao et al. opted for a liquid approach and used citric acid and H_2_O_2_ to simultaneously dissolve the transition metals within LiNi_1/3_Co_1/3_Mn_1/3_O_2_ and generate a chelating agent, which could be subsequently used as a precursor for resynthesizing NCM.^[3]^ Unfortunately, in this approach, the binder and carbon additives are dissolved in *N*‐methylpyrrolidone (NMP) prior to immersing the active material in citric acid, which raises concerns for large‐scale production due to the toxicity of the employed solvent. It should also be noted that, beyond citric acid, a wide range of alternative acids can be used for the active material dissolution, such as HNO_3_,[^4^, ^5^, ^6^] HCl,[^6^, ^7^, ^8^, ^17^] H_2_SO_4_,[^6^, ^9^, ^10^, ^11^] or organic acids.[^12^, ^21^] One can also use bacteria (e. g., *Thiobacillus ferrooxidans*) for the leaching process, which offer high leaching efficiencies and low costs; however, these solutions are not very robust, and the leaching environment must be strictly controlled.[^13^, ^14^] Furthermore, when recycling multicomponent materials like NCM, the ability to individually separate the elemental components is highly beneficial. Li et al. combined oxygen‐free roasting and wet magnetic separation for the separation of Li and cobalt from spent LiCoO_2_.^[22]^ However, this technique is not appropriate for more complex systems like NCM. For such systems, solvent extraction from the leached liquor is the most effective way to separate the transition metals form each other.^[18]^ Unfortunately, the used solvents are still extremely expansive.[^9^, ^23^] Individual precipitation of each component is an interesting and cheap alternative, although the efficiency is lower. Additives like NaOH,^[4]^ LiOH,^[24]^ KF,^[25]^ NH_3_,^[26]^ Na_2_CO_3_,[^4^, ^26^] C_2_H_2_O_4_,^[19]^ and NaClO^[6]^ are often used to individually precipitate the desired salts. In the work of Paulino et al., the authors used NaOH to precipitate Mn(OH)_2_ and Co(OH)_2_ and afterwards KF to obtain LiF.^[25]^

In this work, we present an alternative approach for the recycling of Li_1.02_(Ni_0.8_Co_0.1_Mn_0.1_)_0.98_O_2_ (NCM‐811) that is facile, rapid, and scalable. The initial decomposition of NCM‐811 in a CO_2_ atmosphere produces Li_2_CO_3_, which is easily extracted through dissolution in water. The NCM‐811 precursors are then synthesized from the remaining transition metal oxide by converting it into transition metal oxalates using oxalic acid. Given the different solubilities of the oxalates in an acidic environment, this recycling process also enables the individual separation of the transition metals through a careful control of the pH. The crystallinity and morphology of the intermediates throughout the recycling process were monitored by X‐ray diffraction (XRD) and scanning electron microscopy (SEM), respectively. Meanwhile, inductively coupled plasma optical emission spectroscopy (ICP‐OES) and energy dispersive X‐ray spectroscopy (EDX) were used to confirm the elemental composition and verify the purity of the intermediates. Additionally, X‐ray photoelectron spectroscopy (XPS) and carbon analysis were employed to probe for impurities as well. Finally, the electrochemical performance of the recycled NCM‐811 was assessed using a half‐cell configuration, demonstrating that the recycled material can deliver up to 80 % of the initial capacity of pristine NCM‐811. Most importantly, this versatile recycling approach represents an efficient and environmentally benign alternative to existing recycling methods for Li‐ion battery electrode materials.

## Results and Discussion

**Figure 1:**
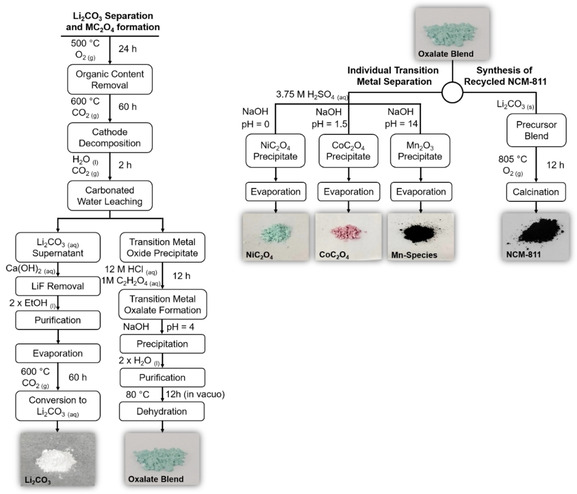
Flow diagram of the employed recycling approach for NCM‐811. During the recycling process each component of the active material can be individually separated, or the active material can be regenerated using the oxalate blend and the Li_2_CO_3_.

In this work, a process for the individual separation of lithium carbonate and the transition metals in NCM‐811, as well as the generation of recycled NCM‐811 is described (Figure 1). The recycling process involves the decomposition of a composite electrode containing NCM‐811, polyvinylidene fluoride (PVDF), and carbon, followed by the subsequent isolation of Li_2_CO_3_ and the formation of a transition metal oxalate blend. From here, the transition metals can either be individually separated from the oxalate blend or the blend can be mixed in the proper stoichiometric ratio with Li_2_CO_3_ to generate recycled NCM‐811. Most importantly, the desired component materials can be obtained with appreciable yields and high purities (see below).

### Li_2_CO_3_ separation and oxalate formation

Given that current separation techniques enable the thorough separation of casing parts from the active material and current collectors,[^5^, ^27^, ^28^, ^29^] the pristine NCM‐811 film is first separated from the aluminum substrate and used as the starting material for the recycling process. From here, the mixture of NCM‐811, PVDF binder and carbon additives is thermally decomposed in a furnace with a flowing atmosphere. The mixture is first heated to 500 °C in an O_2_ atmosphere in order to decompose the organic binder and subsequently heated to 600 °C in a CO_2_ atmosphere to decompose the NCM‐811 into a transition metal oxide and Li_2_CO_3_ [Equation (1)]. The thermal decomposition in an oxygen atmosphere should also remove electrolyte decomposition products, however, which were absent in our starting material. During cycling, a cathode electrolyte interphase (CEI) is always formed, which consists of organic as well as inorganic products. We assume that the binder material and its decomposition products (e. g., LiF, see below), which are formed during the heating step, simulate similar conditions as in cycled NCM.

(1) $$2\;\mathrm{LiNi}{}_{0.8}\mathrm{Co}{}_{0.1}\mathrm{Mn}{}_{0.1}O{}_{2}+\mathrm{CO}{}_{2}\to \mathrm{Li}{}_{2}\mathrm{CO}{}_{3}+2\;\mathrm{Ni}{}_{0.8}\mathrm{Co}{}_{0.1}\mathrm{Mn}{}_{0.1}O+0.5\;O{}_{2}$$

**Figure 2:**
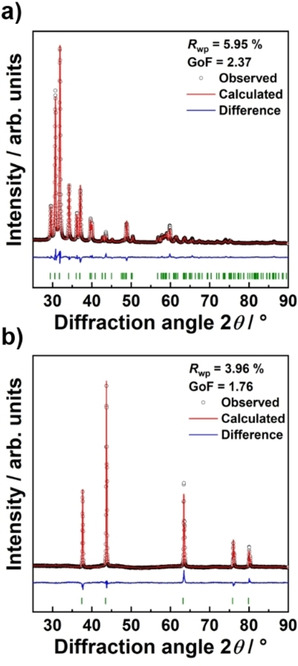
Diffraction patterns and corresponding Rietveld refinements of (a) lithium carbonate and (b) the separated transition metal oxide solid solution.

Research on binding CO_2_ by lithium compounds has been reported as a potential sequestration process.[^30^, ^31^] As can be seen from Equation (1), the presented recycling process also offers the possibility to sequester CO_2_ in the form of Li_2_CO_3_. By using waste CO_2_ from industrial processes, the carbon footprint of the recycling process might be reduced even further. The Li_2_CO_3_ and the transition metal oxide were easily separated through the addition of water to dissolve Li_2_CO_3_. Afterwards, the solid solution of transition metal oxides was extracted from the aqueous Li_2_CO_3_ supernatant by centrifugation, while the Li_2_CO_3_ was isolated by evaporation. However, a minor LiF impurity fraction was detected, stemming from the decomposition of PVDF. The yield of lithium after the thermal decomposition of NCM‐811 within a CO_2_ atmosphere is approximately (75.2±0.1) %. Given that fluorides have been shown to negatively influence the structural properties of NCM, it is necessary to remove any fluoride contaminants from the precursors. Consequently, the Li_2_CO_3_ is first dissolved in H_2_O and mixed with a saturated aqueous solution of Ca(OH)_2_. Li_2_CO_3_ and LiF are converted into LiOH and insoluble CaF_2_, which can be easily separated. After the solvent evaporation, the obtained LiOH is transformed back into Li_2_CO_3_ by heating to 600 °C within a flowing CO_2_ atmosphere. As indicated by the Rietveld refinement in Figure 2a and the lattice parameters in Table S1, which are in good agreement with other publications, high purity Li_2_CO_3_ is obtained.^[32]^ The pristine white color of the Li_2_CO_3_ powder, the cubic morphology of the crystals and the accompanying elemental analysis further corroborate the purity of the separated phase (Figure S1). Moreover, essentially zero transition metal content was detected in the Li_2_CO_3_ by ICP and EDX (Figure S1c). Furthermore, a precise determination of the carbon content by the Dumas method revealed a value of approximately (15.9±0.1) wt %, which is in good agreement with the theoretical value of 16.3 wt % (Table S2).

The diffraction pattern of the NCM‐811 decomposition product following the separation of the Li_2_CO_3_ is shown in Figure 2b. A solid solution of the divalent transition metal oxides is observed, exhibiting no residual reflections of NCM‐811. The transition metal oxide solid solution was refined with a cubic rock‐salt phase (space group $Fm\bar{3}m$ ). All structural parameters extracted from the Rietveld refinement are provided in the Supporting Information, Table S3. The purity of the transition metal oxide phase was additionally corroborated by the Dumas method, which indicated that essentially no residual carbon from the binder or the carbon additives remain (Table S2). Moreover, EDX and ICP data show that the nominal ratio of the transition metals is maintained (Figure S2). The yield of the transition metal oxide after decomposition is (96.6±0.1) %.

In the next step, the previously obtained oxide is converted into transition metal oxalates through the dissolution of the oxide in 12 M HCl, followed by the addition of 15 mL of 1 M oxalic acid. Once the transition metal oxalates are in solution, a rapid increase of the pH to a value of 4 leads to the precipitation of an oxalate blend in the same stoichiometry as the starting material, which is confirmed by ICP and EDX (Figure S2). Given that the oxalates tend to be hygroscopic, it is likely that the dihydrates are formed, which was confirmed by the Dumas method. The measured weight fraction of carbon is (12.8±0.1) wt %, which is closer to the theoretical value of the dihydrate than to the anhydrous oxalate (Table S2). Based on the transition metal oxalate dihydrate, the yield of the reaction is approximately (88.2±0.1) %. It should be noted that the supernatant exhibited a greenish hue after the extraction of the precipitated oxalates, indicating that the precipitation was not fully complete. However, the addition of excess oxalic acid did not result in further precipitation.

### Individual transition metal separation

Given the variety of NCM compositions that exhibit good performance as cathode materials in lithium‐ion batteries (e. g., LiNi_0.33_Co_0.33_Mn_0.33_O_2_, LiNi_0.5_Co_0.2_Mn_0.3_O_2_, LiNi_0.6_Co_0.2_Mn_0.2_O_2_),[^33^, ^34^, ^35^] the ability to isolate each transition metal component during the recycling process would allow for application‐dependent compositional tailoring in the synthesis of recycled NCM. The challenging task is, of course, the individual separation of the transition metals. Generally, the transition metals are isolated using a solvent extraction technique,[^9^, ^36^] however this is tedious, as well as time‐ and cost‐intensive. Herein, the transition metals can be easily separated by exploiting the different solubilities of the transition metal oxalates. Upon dissolving the synthesized transition metal oxalate mixture in H_2_SO_4_, NiC_2_O_4_ can immediately be separated from the solution (Figure 1). Through the addition of NaOH, the pH can be slowly and precisely elevated to initiate the individual precipitation of both remaining transition metal species. By increasing the pH to approximately 1.5, the precipitation of CoC_2_O_4_ is initiated. It should be noted that after the separation of nickel and cobalt, only Mn remains in the supernatant and could already be used for syntheses. However, for the determination of impurities also the Mn fraction was precipitated. Further increasing the pH of the supernatant to nearly 14 results in the complete precipitation of a mixture of oxidized manganese species. Diffraction patterns of the individual transition metal fractions can be found in the Supporting Information, Figure S3. The nickel and cobalt patterns exhibit reflections from both the anhydrous and the dihydrate oxalates, while the pattern of the manganese fraction exhibited a rather low intensity with fairly broad reflections, thus complicating the phase identification. The low crystallinity of the manganese fraction likely stems from the high pH required for the separation. Nevertheless, the pattern can be primarily assigned to Mn(OH)_2_ and MnC_4_O_2_, however the black color of the precipitate could arise from the presence of amorphous MnO_2_.[^37^, ^38^]

**Figure 3:**
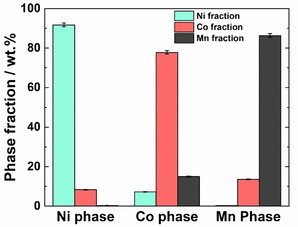
Elemental analysis by EDX showing the individual separation of the transition metals from the transition metal oxalate blend achieved through the tuning of the pH. Measurements were conducted at multiple regions on the sample.

The purity of the individually separated transition metal fractions was determined by EDX (Figure 3). The major metal fraction for NiC_2_O_4_ and MnC_2_O_4_ is >85 wt %. However, the major metal fraction for CoC_2_O_4_ is approximately 78 wt %. The minority fractions in the transition metal phases represent the remaining Ni/Co/Mn content present in the main fraction. The slightly lower purity of the cobalt fraction arises from the precise control required over the pH for the transition metal separation. In other words, a small amount of nickel remains after the removal of the NiC_2_O_4_ fraction and some MnC_2_O_4_ is also precipitated upon elevating the pH to fully remove the CoC_2_O_4_ fraction. As can be seen in Figure 3, it is obvious that further investigations regarding the optimal separation conditions are necessary. Future experiments should aim for optimizing the pH value, the reaction time and the used acid. Additionally, the use of additives, which influence the solubility of the transition metal oxalates is a viable option. The obtained transition metal oxalates exhibit very distinct colors (see Figure 1), which are in good agreement with the previously reported physical properties for these compounds.[^37^, ^39^, ^40^]

### Synthesis of recycled NCM‐811

**Figure 4:**
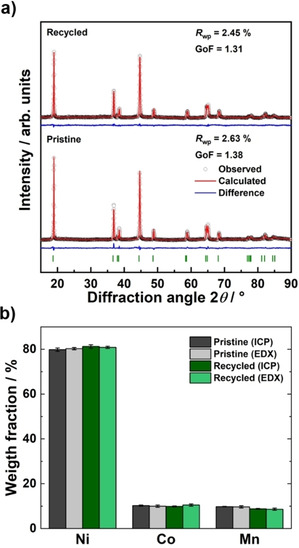
(a) Experimental XRD patterns for the pristine and recycled NCM‐811. All diffraction maxima could be indexed to the layered *R* $\overset{-}{3}$ *m* structure with no observable impurities. The Rietveld refinement revealed that the recycled NCM‐811 exhibits slightly higher site mixing compared to the pristine NCM‐811. (b) Comparison of the elemental compositions for the pristine and the recycled NCM‐811, as determined by ICP‐OES and EDX measurements. The EDX measurements were conducted at multiple regions on the sample and for the ICP measurements the average of three measurements was calculated. The experimental composition of the recycled NCM‐811 is in good agreement with the nominal composition of Li_1.02_(Ni_0.8_Co_0.1_Mn_0.1_)_0.98_O_2_, as also found in the pristine NCM‐811.

Beyond the individual separation of the transition metals, the transition metal oxalate blend and the obtained Li_2_CO_3_ can be mixed using the appropriate stoichiometric quantities with a 5 mol % excess of Li (TM/Li=1 : 1.05) and treated at 805 °C in a flowing O_2_ atmosphere to afford recycled NCM‐811 (Figure 1). Following the recycling process, the recycled NCM‐811 exhibits the expected layered NCM structure (Figure 4a). No additional side phases were observed, confirming the successful synthesis of NCM‐811. Furthermore, the lattice parameters are in good agreement with the refined values obtained for the pristine NCM‐811 (see Supporting Information, Table S4 and S5). An important factor affecting the electrochemical performance in NCM is the cation site‐disorder. Due to similar ionic radii of Li^+^ and Ni^2+^, Ni^2+^ can move into the lithium layer and occupy the 3*b* Wyckoff position, where it blocks Li^+^ diffusion.^[41]^ Notably, a similar degree of site disorder was observed in the recycled material and the pristine NCM‐811 [i. e., (7.9±0.4) %]. As the site‐disorder stems from the calcination procedure applied to the precursor mixture, optimization of the calcination conditions can minimize the resultant disorder.[^42^, ^43^, ^44^]

Given that the morphology of NCM depends on the morphology of the precursors, it is important to monitor the morphology of the used intermediates. The evolution of the particle morphology throughout the recycling process, as determined by SEM, is illustrated in Figure S4. The pristine NCM‐811 particles consist of primary particles with an average diameter of approximately 200 nm, which agglomerate to secondary particles with diameters between 10–20 μm. Upon thermal decomposition into the binary oxides, the particles retain a spherical shape; however, the surfaces become coated with Li_2_CO_3_ (Figure S4b). The extraction of Li_2_CO_3_ using a carbonated water leaching process mildly destabilizes the secondary particles, resulting in an increase in the number of isolated primary particles. In the next step, the formation of the transition metal oxalates leads to a loss of the spherical shape (Figure S4c). The average diameter of the particles within the transition metal oxalate blend is approximately 11 μm. After blending the oxalates with Li_2_CO_3_ and calcining at 805 °C, the secondary particles are partially sintered, exhibiting fairly irregular shapes with an average diameter of approximately 5 μm (Figure S4d).

In addition to the morphology, the elemental composition of NCM‐811 also plays a crucial role in the resulting electrochemical performance. High nickel fractions offer high capacities, while increasing the cobalt and manganese content leads to better electronic conductivity and mechanical stability, respectively.[^45^, ^46^] The composition of the NCM‐811 was investigated before and after the recycling process using ICP‐OES and EDX measurements (Figure 4b). Both techniques reveal that the pristine and recycled NCM‐811 are in good agreement with the nominal composition of Li_1.02_(Ni_0.8_Co_0.1_Mn_0.1_)_0.98_O_2_. However, a slight deficiency of manganese was detected in the recycled NCM‐811, which is likely due to an incomplete precipitation of manganese oxalate during the recycling process. It should also be noted that a low concentration of sodium (2.4 at %) at the particle surface was detected in the XPS spectra, which could stem from the NaOH used to adjust the pH during the oxalate precipitation and could possibly influence the electrochemical performance (Figure S5). While we cannot exclude that a low concentration of sodium ions are also incorporated into the structure, sodium doping of nickel based oxides have been suggested to improve the electrochemical performance of cathode active materials during cycling.[^47^, ^48^, ^49^] No chloride ions were found following the HCl acid route and the acid is therefore not expected to influence the electrochemical behavior.

### Electrochemical performance of recycled NCM‐811

**Figure 5:**
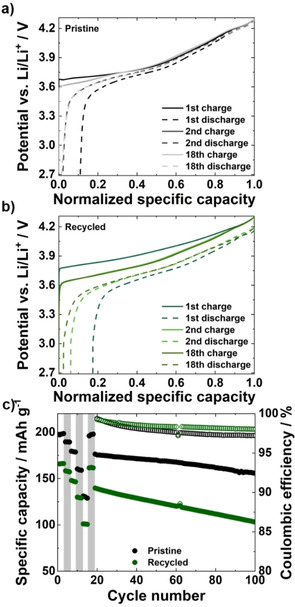
Comparison of the potential profile of the 1st, 2nd, and 18th cycle for a) pristine NCM‐811 and b) recycled NCM‐811. The cells are cycled at 0.1 C in the voltage range of 2.7 to 4.3 V vs. Li^+^/Li. c) Comparison of the rate capability and the long‐term stability of pristine and recycled NCM‐811. During the rate test, the cells were cycled at 0.1, 0.25, 0.5, 1, and 2 C for three cycles each. The cells were then cycled at 0.1 C for three more cycles before the stability test was run at 0.5 C.

The electrochemical performance of the recycled NCM‐811 was investigated with rate capability tests followed by long‐term stability tests using a coin cell architecture. Figure 5a,b shows the potential profiles for the cells constructed with both the pristine and recycled NCM‐811 with increasing cycle number. For the pristine material, the charge plateau starts at 3.7 V vs. Li^+^/Li, whereas for the recycled material the onset is at 3.8 V. Notably, the capacity loss in the first cycle is much larger in the case of the recycled NCM‐811 cell than for the pristine NCM‐811 cell (17.4 and 11.6 %, respectively), which suggests that the recycled material possesses a higher surface area. During the first cycle, CEI formation consisting of electrolyte decomposition and cathode surface passivation takes place, which results in some irreversible capacity loss.[^50^, ^51^, ^52^] The level of the aforementioned effects depends on the surface area of the cathode active material and thus, larger surface areas could lead to larger capacity losses. Further investigations, such as Brunauer‐Emmett‐Teller (BET) analysis, are needed to prove this hypothesis.

Figure 5c shows the rate‐dependent specific discharge capacities over 100 cycles. The recycled NCM‐811 achieves up to 166 mAh g^−1^ in the first cycle at 0.1 C, which corresponds to 84 % of the capacity of the pristine NCM‐811 electrode. The lower capacities observed in the recycled NCM‐811 cell can most likely be attributed to the previously mentioned undefined particle morphology, given that the morphology strongly influences the intercalation behavior. Well‐defined spherical particles have already been shown to intercalate lithium‐ions more efficiently, thereby achieving higher lithium utilization.^[53]^ Contrastingly, regarding the long‐term stability, the recycled NCM‐811 exhibits more stable coulombic efficiencies, exceeding 98 % throughout.

## Conclusions

Herein, we have demonstrated a rapid and facile process for the recycling of LiNi_1−*x*−*y*_Co_*x*_Mn_*y*_O_2_ (NCM) cathode materials for Li‐ion batteries. The recycling process offers the possibility to reuse the active elements in the cathode material without the need for complex separation processes. First, spent NCM is decomposed into Li_2_CO_3_ and Ni_0.8_Co_0.1_Mn_0.1_O. By a simple dissolution process, the oxide is converted into an oxalate, which serves as a precursor for fresh NCM‐811. X‐ray diffraction, inductively coupled plasma optical emission spectroscopy, and energy‐dispersive X‐ray spectroscopy revealed the excellent purity of the Li_2_CO_3_, the individually separated transition metal fractions, as well as the recycled NCM‐811. Given that a variety of different NCM compositions are currently being employed in Li‐ion batteries, another crucial factor is the individual separation of the transition metals. This approach offers the possibility to efficiently separate the transition metals, thereby enabling the synthesis of a variety of NCM compositions independent of the starting composition. For the separation of the transition metals components, a simple pH‐dependent precipitation process was used. Furthermore, the electrochemical performance of half‐cells containing the recycled NCM‐811 was investigated and showed an initial specific capacity of 166 mAh g^−1^ at 0.1 C, which is approximately 84 % of the pristine NCM electrode capacity. Nevertheless, optimization of the NCM‐811 particle morphology could be performed to further enhance the electrochemical performance. Ultimately, the presented method offers a rapid, facile, and scalable alternative to existing recycling approaches for Li‐ion battery electrode materials.

## Experimental Section

**Recycling procedure**: The recycled NCM‐811 was prepared through the decomposition of Li_1.02_(Ni_0.8_Co_0.1_Mn_0.1_)_0.98_O_2_ (BASF SE). First, fresh electrodes of NCM‐811 were prepared by mixing 90 wt % NCM with 5 wt % SuperPLi (Timcal) and 5 wt % Solef PVDF 5130 (BASF SE). The electrode slurry was stirred for 12 h to ensure a homogenous distribution of all components. Afterwards, a 300 μm thick film was cast on aluminum foil using a doctor blade. The electrode sheet was dried for 24 h in vacuum and subsequently pressed with a roll press (MSK‐HRP‐04, MTI Corporation) to a thickness of 60 μm. All described steps were performed under an inert atmosphere or vacuum to prevent the exposure to air and avoid side reaction of NCM with moisture. Current processes enable the separation of cell parts at such a level that only a mixture of active materials, binder and carbon remain for the separation.[^5^, ^27^, ^28^] To simulate this step, the active material/binder/carbon film was scratched from the aluminum current collector. The mass of the removed material was approximately 1.46 g. The organic material was first decomposed in a furnace by heating to 500 °C in a flowing (500 sccm) O_2_ atmosphere and afterwards the active material was decomposed by heating to 600 °C in a flowing CO_2_ (500 sccm) atmosphere for 60 h. Reaction times shorter than 60 h resulted in only partial conversion of NCM into Li_2_CO_3_ and Ni_0.8_Co_0.1_Mn_0.1_O. After decomposition, 1.37 g of the resulting blend (i. e., transition metal oxide and Li_2_CO_3_) was dispersed in 150 mL distilled water to separate the Li_2_CO_3_ from the transition metal oxides. To increase the solubility of Li_2_CO_3_, CO_2_ was also bubbled in the solution. After centrifuging, a solution containing Li_2_CO_3_ and a precipitate of transition metal oxides were obtained (0.97 g). Upon evaporating the water from the Li_2_CO_3_ solution, LiF contaminated Li_2_CO_3_ was obtained (352.50 mg), which arises from the PVDF binder used in the cathode composite. LiF was removed by dissolving the obtained Li_2_CO_3_ in water and mixing the solution with a saturated aqueous Ca(OH)_2_ solution to convert LiF into CaF_2_, which can be easily removed from the solution due to the low water solubility. Since Li_2_CO_3_ is also partly transformed into LiOH during this step, the Li_2_CO_3_/LiOH mixture was again heated to 600 °C in a CO_2_ flow to convert the product back to Li_2_CO_3_. From here, the transition metal oxalates were synthesized to be used as precursors for the subsequent NCM synthesis. Therefore, 0.97 g of the transition metal oxide were dissolved in 50 mL concentrated HCl (HCl+10 vol % H_2_O_2_) and 15 mL of 1 m oxalic acid was added to the solution. In order to precipitate the oxalates, the pH was increased through the addition of NaOH to the solution. By rapidly increasing the pH to 4, a blend of NiC_2_O_4_, CoC_2_O_4_, and MnC_2_O_4_ was obtained. After the precipitation was finished, the oxalate blend was washed three times with water and dried at 80 °C in vacuum for 12 h. Finally, stoichiometric amounts of the transition metal oxalate and Li_2_CO_3_ (5 mol % of excess of Li were used to compensate the Li evaporation during synthesis) were mixed and heated to 805 °C in a flowing O_2_ atmosphere (200 sccm) to prepare recycled NCM. After the synthesis, the recycled NCM was directly transferred into an argon‐filled glovebox.

The separation of the transition metals was achieved as follows: first, the synthesized mixed transition metal oxalate blend was dissolved in 15 mL H_2_SO_4_ (3.75 mol L^−1^; pH: −0.5). At this point, NiC_2_O_4_ already precipitated and was separated from the solution by centrifugation. After the separation of NiC_2_O_4_, the pH was increased through the addition of NaOH. At a pH of approximately 1.5, CoC_2_O_4_ precipitated, which was also separated by centrifugation. Finally, by further increasing the pH to nearly 14, a mixture of several manganese species (i. e., MnC_2_O_4_ and Mn(OH)_2_) precipitate. All precipitates were centrifuged and washed twice with distilled water.

**XRD**: Powder XRD was carried out using an Empyrean diffractometer (PANalytical, Netherlands) with Cu_Kα_ radiation in Bragg–Brentano *θ*–*θ* geometry using a PIXcel3D area detector with 255 measuring channels. XRD data were collected in the range 2*θ*=10–90°. In order to minimize the effect of X‐ray fluorescence of the transition metals, the PHD upper and lower values were set to 70 and 49, respectively.

**Rietveld analysis**: Rietveld refinements were carried out using the TOPAS software package.^[54]^ The average crystal structure of Li_1.02_(Ni_0.8_Co_0.1_Mn_0.1_)_0.98_O_2_ (NCM‐811) was refined using the rhombohedral $R\bar{3}m$ space group (no. 166) as a starting model. For decomposition products, namely Li_2_CO_3_ and Ni_0.8_Co_0.1_Mn_0.1_O, the space group *C*2/*c* (No. 15) and $Fm\bar{3}m$ (No. 202) were used, respectively. Fit indicators: *R*_wp_, and goodness‐of‐fit were used to assess the quality of the refined structural models.^[55]^ The following parameters were initially refined: (1) scale factor, (2) background coefficients, (3) sample displacement, (4) peak shape, which was modeled using a modified Thomson‐Cox‐Hastings pseudo‐Voigt function,^[56]^ (5) lattice constants, (6) fractional atomic coordinates, and (7) isotropic atomic displacement parameters. The site mixing of Li^+^ and Ni^2+^ was also determined through the refinement of the occupancy of Ni on the *3b* Wyckoff position of Li.

**ICP‐OES**: ICP measurements were conducted using a Varian 720ES instrument with an axial flare, Echelle‐optics, and a CCD detector. Approximately 10 mg of each sample were dissolved in 10 ml of 2 m HCl. The samples were measured with a power of 1.2 kW and the measurement time was 20 s.

**SEM/EDX**: SEM and EDX measurements were conducted with a “Merlin” instrument from Zeiss. For imaging, the accelerating was 5 kV and the current was 120 pA. For the elemental EDX analysis an X‐Max detector from Oxford Instruments was used. The accelerating voltage was increased to 15 kV and the accelerating current 700 pA to obtain elemental maps with sufficient intensity.

**Cell assembly and electrochemical testing**: Electrodes of recycled NCM were made as described above. After the electrode sheet preparation, circular electrodes with 12 mm in diameter were punched out. The cells were assembled as CR2032 type coin cells (MTI Corporation) in an Ar‐filled glovebox (Glovebox Systemtechnik). The cells were tested in a half‐cell configuration using metallic lithium (Rockwood Lithium GmbH) as the anode material and Whatman® GF/A Glass microfiber filters as the separator. 100 μL LP50 (Sigma Aldrich) were used as the electrolyte, which is a mixture of LiPF_6_ dissolved in ethylene carbonate and ethyl methyl carbonate (50 : 50 *w*/*w*). The prepared cells were sealed with a crimper (MTI). The rate capability and the long‐term stability was measured for each cell. Therefore, the cycling protocol contains an initial C‐rate test (0.1, 0.25, 0.5, 1.0, 2.0, and again 0.1 C; each C‐rate was measured for three cycles) and a long‐term test (0.5 C for 90 cycles) was also performed.

## Conflict of interest

The authors declare no conflict of interest.

## Supporting information

As a service to our authors and readers, this journal provides supporting information supplied by the authors. Such materials are peer reviewed and may be re‐organized for online delivery, but are not copy‐edited or typeset. Technical support issues arising from supporting information (other than missing files) should be addressed to the authors.

Supplementary
