# Supplementary material for: A Rapid and Facile Approach for the Recycling of High‐Performance LiNi1−x−yCoxMnyO2 Active Materials
Source: ChemSusChem. 2020 Sep 10;14(1):441–8. doi: 10.1002/cssc.202001915 (PMC7821189; doi:10.1002/cssc.202001915)
Supplement: Supplementary file 1 — Supplementary [file CSSC-14-441-s001.pdf]

ChemSusChem

Supporting Information

A Rapid and Facile Approach for the Recycling of High-Performance $LiNi_{1-x-y}Co_xMn_yO_2$ Active Materials

Jan O. Binder, Sean P. Culver, Wolfgang G. Zeier,* and Jürgen Janek*© 2020 The Authors. Published by Wiley-VCH GmbH. This is an open access article under the terms of the Creative Commons Attribution License, which permits use, distribution and reproduction in any medium, provided the original work is properly cited.

Experimental

Dumas method. The carbon analysis was carried out with an EC Vario Cube (Elementar). For each analysis, three measurements of each sample were conducted to ensure the reliability of the measurement.

X-ray photoelectron spectroscopy (XPS). XPS analysis of the pristine and recycled NCM-811 were performed using a PHI 5000 Versaprobe II Scanning ESCA Microporbe (Physical Electronics). The pressure in the chamber was controlled to be below 10^{-8} mBar and a monochromatic Al anode was used as X-ray source. The pass energy for a survey spectra was chosen to be 23.5 eV. The C 1 s line at 284.8 eV was applied to calibrate the spectra. The spectra were analyzed with the CasaXPS software.

Table S1. Refined structural parameters for lithium carbonate after purification. The B_{eq} for Li is fixed set to 6, as the X-ray form factor for Li is too low to obtain reasonable atomic displacement parameters.

Li_2CO_3 structure from powder X-ray diffraction data (space group *C*2/*c*); λ = 1.5406 Å; *a* = 8.3622(3) Å, *b* = 4.9774(2) Å, *c* = 6.1983(3) Å, *β* = 114.696(2) °; Fit residuals (R_{wp}, R_{exp}, GoF): 5.95%, 2.51%, 2.37						
Atom	Wyckoff Site	x/*a*	y/*b*	z/*c*	Occ.	B_{eq} / Å^2
Li1	8*f*	0.3035	0.0516	0.1656	1	6
O1	8*f*	0.3529(3)	0.4415(4)	0.1889(3)	1	5.15(6)
O2	4*e*	0.0	0.31(5)	0.25	1	5.8(1)
C1	4*e*	0.0	0.036(1)	0.25	1	5.5(1)

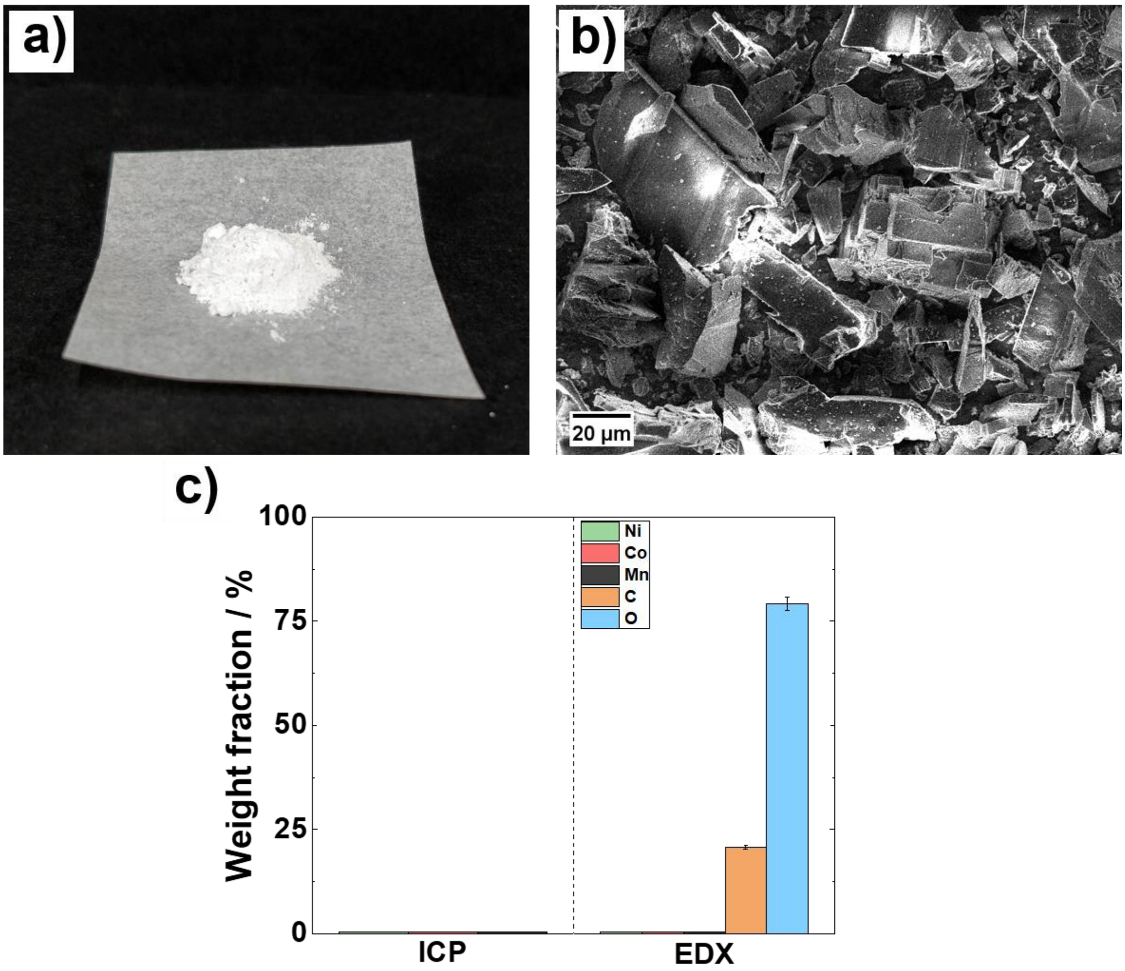

Figure S1. a) Image of the separated Li_2CO_3 powder. The pure white color is an indication for the phase purity of this phase. b) SEM image of the separated Li_2CO_3, which exhibits cubic crystals. The shape of the cubes leads to changed reflection intensities due to preferred orientation. c) Elemental composition of the Li_2CO_3 powder determined by ICP-OES (left) and EDX measurements (right). As can be seen from this data, the concentration of transition metals in 10 mg Li_2CO_3 powder dissolved in 5 ml 4M HCl solution is almost 0. In the EDX measurements no transition metals can be detected at all and the ratio of C and O is very close to the theoretical value (20:80 wt.%).

Table S2. Comparison of the measured carbon contents for Li_2CO_3, $Ni_{0.8}Co_{0.1}Mn_{0.1}O$ and Ni/Co/Mn oxalate blend by the Dumas method and their theoretical values. The value for Li_2CO_3 is already corrected for 8.6 wt.% of LiF.

Sample	Theoretical carbon content / wt.%	Measured carbon content / wt.%
Li_2CO_3	16.25	15.86(8)
$Ni_{0.8}Co_{0.1}Mn_{0.1}O$	-	0.17(4)
Ni/Co/Mn oxalate blend	16.41	12.84(8)
Ni/Co/Mn oxalate dihydrate blend	13.17	

Table S3. Refined structural parameters for the solid solution of the transition metal oxide.

$Ni_{0.8}Co_{0.1}Mn_{0.1}O$ structure from powder X-ray diffraction data (space group $Fm\bar{3}m$);<br>λ = 1.5406 Å<br>*a* = 4.1569(1) Å<br>Fit residuals (R_{wp}, R_{exp}, GoF): 3.96%, 2.25%, 1.76						
Atom	Wyckoff Site	*x*/*a*	*y*/*b*	*z*/*c*	Occ.	B_{eq} / Å^2
Ni1	4*b*	0.50	0.50	0.50	0.8	1.34(4)
Co1	4*b*	0.50	0.50	0.50	0.1	1.34(4)
Mn1	4*b*	0.50	0.50	0.50	0.1	1.34(4)
O1	4*a*	0.0	0.0	0.0	1	1.5

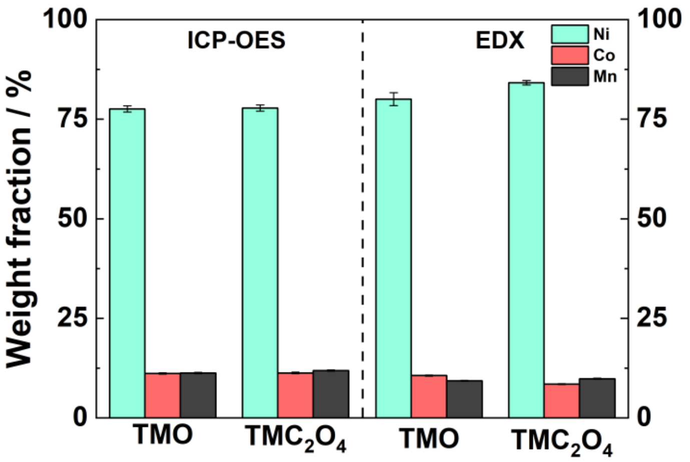

Figure S2. Comparison of the elemental composition of the solid solution of the transition metal oxides and oxalate blend obtained by ICP-OES and EDX measurements. The ratio of the transition metals in both compounds is nearly 8:1:1 for Ni:Co:Mn.

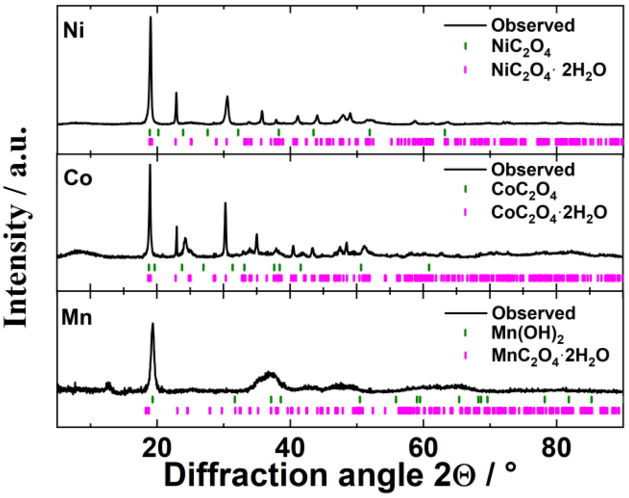

Figure S3. Diffraction patterns of the separated transition metal phases by tuning the pH with NaOH after the oxalate blend was dissolved in H_2SO_4. For the Ni- and Co phases, only the anhydrous and the dihydrate oxalates can be found. Meanwhile, for the Mn phase, a clear identification of the phases is quite difficult due to the low intensities and breadth of the reflections, however it is likely that this fraction consists of $Mn(OH)_2$ and $MnC_2O_4{\cdot}2H_2O$.

Table S4. Refined structural parameters for the pristine NCM-811. The B_{eq} value for Li was fixed to 5, as the X-ray form factor for Li is too low to obtain reasonable atomic displacement parameters. For the determination of the occupancy of Ni1, Ni2, Li1 and Li2 the following constraints were used: $O_{Ni1}=O_{Li2}=x$, $O_{Ni2}=0.8-x$, $O_{Li1}=1-x$.

$LiNi_{0.8}Co_{0.1}Mn_{0.1}O$ structure from powder X-ray diffraction data (space group $R\bar{3}m$);
λ = 1.5406 Å
a = 2.8722(1) Å, *c* = 14.2067(4)
Fit residuals (R_{wp}, R_{exp}, GoF): 2.63%, 1.90%, 1.38

Atom	Wyckoff Site	x/*a*	y/*b*	z/*c*	Occ.	B_{eq} / $Å^2$
Li1	3*b*	0	0	0.50	0.924(1)	5
Ni1	3*b*	0	0	0.50	0.076(1)	5
Ni2	3*a*	0.0	0.0	0.0	0.724(1)	1.39(4)
Li2	3*a*	0.0	0.0	0.0	0.076(1)	1.39(4)
Co1	3*a*	0.0	0.0	0.0	0.1	1.39(4)
Mn1	3*a*	0.0	0.0	0.0	0.1	1.39(4)
O1	6*c*	0.0	0.0	0.2537(1)	1	1.85(6)

Table S5. Refined structural parameters for recycled NCM-811. The $B_{eq,}$ value for Li was fixed to 5, as the X-ray form factor for Li is too low to obtain reasonable atomic displacement parameters. For the determination of the occupancy of Ni1, Ni2, Li1 and Li2 the following constraints were used: $O_{Ni1}=O_{Li2}=x$, $O_{Ni2}=0.8-x$, $O_{Li1}=1-x$.

$LiNi_{0.8}Co_{0.1}Mn_{0.1}O$ structure from powder X-ray diffraction data (space group $R\bar{3}m$);
λ = 1.5406 Å
a 2.8705(1) Å, *c* 14.1893(5)
Fit residuals (R_{wp}, R_{exp}, GoF): 2.45%, 1.87%, 1.31

Atom	Wyckoff Site	x/*a*	y/*b*	z/*c*	Occ.	B_{eq} / $Å^2$
Li1	3*b*	0	0	0.50	0.921(1)	5
Ni1	3*b*	0	0	0.50	0.079(1)	5
Ni2	3*a*	0.0	0.0	0.0	0.721(1)	1.46(4)
Li2	3*a*	0.0	0.0	0.0	0.079(1)	1.46(4)
Co1	3*a*	0.0	0.0	0.0	0.1	1.46(4)
Mn1	3*a*	0.0	0.0	0.0	0.1	1.46(4)
O1	6*c*	0.0	0.0	0.2537(1)	1	1.87(6)

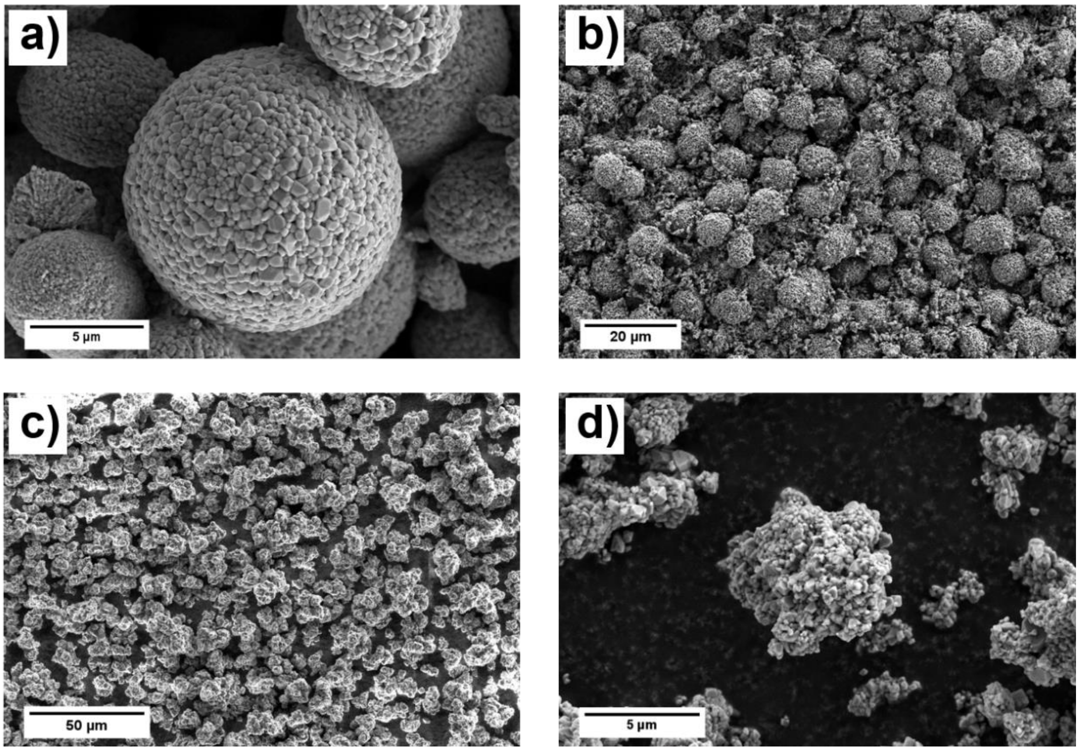

Figure S4. SEM micrographs depicting the variation of the morphology during the recycling approach for each intermediate. After decomposition of (a) the pristine NCM-811 to (b) the transition metal oxide solid solution and Li_2CO_3, the Li_2CO_3 can be easily dissolved in water. (c) The spherical shape is only partially recovered after the formation of the transition metal oxalates, which is also visible in (d) the recycled NCM-811.

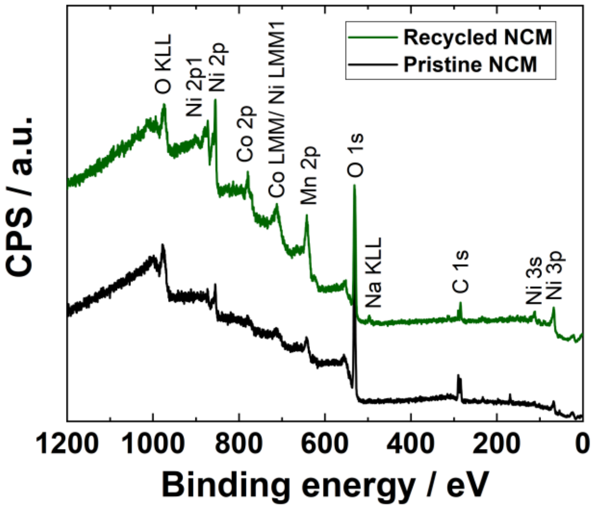

Figure S5. Comparison of the XPS spectra of pristine and recycled NCM. There is no shift of the transition metal signals, indicating that there is no change in the oxidation state. In the spectra of recycled NCM, additional signals of sodium can be found, which originates from the NaOH that remains after the synthesis.
